# Regional & Topical Anaesthesia of Upper Airways

**Published:** 2009-12

**Authors:** Nibedita Pani, Shovan Kumar Rath

**Affiliations:** 1 Professor, Department of Anaesthesiology, SCB Medical College, Cuttak-753007, Orissa; 2 P G Student, Department of Anaesthesiology, SCB Medical College, Cuttak-753007, Orissa

**Keywords:** Awake intubation, Fibre optic intubation, Laryngoscopy, Topical anaesthesia, Local anaesthetics, Nerve block

## Abstract

**Summary:**

A combination of techniques are required to adequately anaesthetise upper airway structures for awake intubation. The widest coverage is provided by the inhalational technique. This technique, however, does not always provide a dense enough level of anaesthesia for all patients. Supplementation of this technique with any of the specific nerve blocks is an excellent way to accomplish efficacious anaesthesia for awake inubation. Anaesthetising upper airway is not a difficult skill to master and should be in the armamentarium of all practising anaesthetist.

## Introduction

Airway management is fundamental to the practice of anaesthesia and tracheal intubation is frequently required to ensure adequate airway control, while providing optimal operating conditions. Intubation by direct laryngoscopy is usually satisfactory. However, providing anaesthetic care to the patient with a difficult airway keenly interests anaesthesiologists. Expertise with regional anaesthesia of the airway allows intubation in awake patients with suspected difficult intubation in the presence of certain anatomical variants or airway pathology, where visualization of the glottis by direct laryngoscopy can be difficult or impossible. Furthermore, in the presence of upper airway trauma & cervical spine injury, neck movement must be minimized if neurological injury is to be avoided. Therefore, it is essential that every regional anaesthesiologist be skilled enough to administer general anaesthesia and especially in the management of the difficult airway with the knowledge of recent development in the field of regional anaesthesia of the upper airways.

The mainstay of difficult airway management remains flexible fiberoptic laryngobronchoscopic intubation. In the awake patient fibreoptic intubation maintains a wide margin of safety while producing minimal patient discomfort, but requires adequate local anaesthesia of the airway. Fibreoptic intubation (FOI) is regarded as a safe way of managing some airway problems, particularly anticipated difficulty with direct laryngoscopy^1^–^3^. Several authors have reported that FOI can be achieved with considerable haemodynamic stability^4^–^6^ under local anaesthetic when combined with sedation.

Laryngobronchoscopy in an awake, unprepared patient lead to undesirable elevations in the patient's sympathetic and parasympathetic outflow causing excessive salivation and gag and cough reflexes making intubation difficult, To decide on a proper approach to an awake intubation, one must determine what structures need to be anaesthetized along the two basic routes of intubation (oral or nasal) to facilitate optimal surgical conditions in the context of patient-specific anatomic considerations. Each of these routes has a well-defined pattern of innervation that can be specifically blocked to provide adequate anaesthesia to subdue these reflexes and facilitate intubation.

Although fibreoptic intubation can be done in the unconscious individual, it is particularly suited to the awake patient and, with proper technique, produces minimal discomfort while maintaining a wide margin of safety.^7^–^11^

## Relevant Anatomy

**Fig 1:**
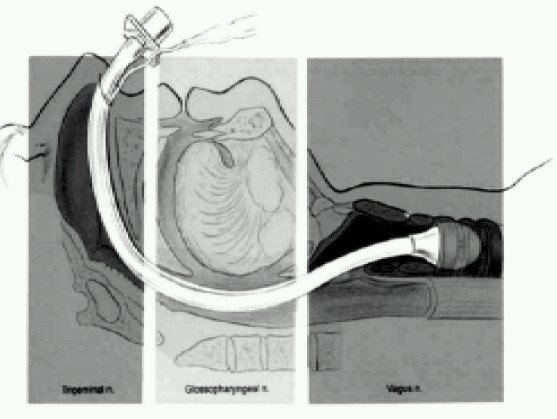
Three major neural pathways supplying sensation to airway structures

Three major neural pathways supply sensation to airway structures (Fig 1)

### 1. Terminal branches of the ophthalmic and maxillary divisions of the trigeminal nerve supply the nasal cavity and turbinates

**Fig 2:**
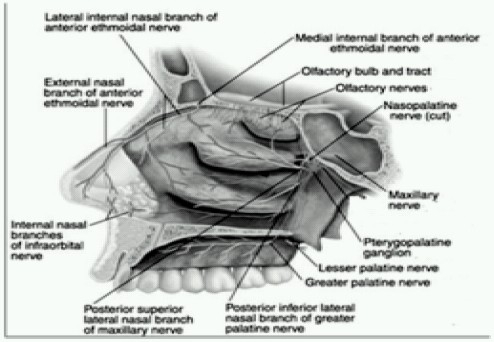
Sensory supply of nose

(Fig 2),^12^ (i.e. the greater and lesser palatine nerves arising from the pterygopalatine ganglion innervate the nasal turbinates and most of the nasal septum.. The anterior ethmoidal nerve arises from the olfactory nerve (CNI) and innervates the nares and the anterior third of the nasal septum.)

### 2. The oropharynx and posterior third of the tongue are supplied by the glossopharyngeal nerve.

(The sensory innervation of the anterior two thirds of the tongue is provide by the trigeminal nerve i. e. lingual branch of the mandibular division. it is not a part of the reflex arcs controlling gag or cough, its blockade is not essential for comfort during FOI.)

### 3. Branches of the vagus nerve innervate the epiglottis and more distal airway structures. (Fig 3)

**Figure 3:**
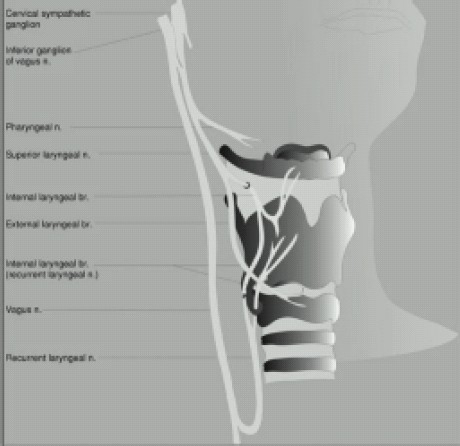
Branches of the vagus nerve innervating the epiglottis and more distal airway structures.

The airway reflexes important for awake intubation

The aforementioned nerves participate in several brainstem-mediated reflex arcs.

**1. Gag reflex** – triggered by mechanical and chemical stimulation of areas innervated by the glossopharyngeal nerve, and the efferent motor arc is provided by the vagus nerve and its branches to the pharynx and larynx.

**2. Glottic closure reflex** – elicited by selective stimulation of the superior laryngeal nerve, and efferent arc is the recurrent laryngeal nerve. – exaggeration of this reflex is called laryngospasm.

**3. Cough** – the cough receptors located in the larynx and trachea receive afferent and efferent fibers from the vagus nerve.

### Techniques for Anaesthetizing the Airway

Even in the hands of the most skilled regional anesthesiologist, blocks are subject to a certain rate of complications or failure^13^–^16^. So Fiberoptic intubation can be performed under a variety of conditions. However, one major decision must be made with every procedure will the patient be intubated while under general anaesthesia, or does the patient need to be awake during intubation? Intubation under general anaesthesia (even with inhalational induction and spontaneous respiration) carries the inherent risk of losing control of the difficult airway. For this reason, many anaesthesiologists, on recognition of a difficult airway, elect to perform an awake intubation using either fiber optic laryngobronchoscopy or awake direct laryngoscopy

### Preparation for Awake Intubation

Adequate sedation & analgesia is important and advantageous in both the anaesthetizing of the airway as well as during the intubation. Agents used for this purpose are generally fall into 2 group: benzodiazepines and opioids. For this purpose, it is best to use short-acting or reversible agents for sedation or agents that do not cause a considerable degree of respiratory depression, i. e midazolam, alfentanil, and fentanyl

Second is Antisialogogues,^17^,^18^ as oral secretions may make visualization via the fiberoptic equipment difficult and may serve as a barrier to effective penetration of local anaesthetic into the mucosa. Glycopyrrolate or atropine may be used^12^,^19^.

## Local anaesthetics

There are three most often used local anaesthetic with or without the use of vasoconstrictors: Cocaine, Benzocaine, & Lidocaine

### Local anaesthetics – benzocaine

A water-insoluble ester that is frequently used in a 20% spray to produce anaesthesia in mucous membranes.

## Local anaesthetics – lidocaine

Lidocaine is probably the local anaesthetic most frequently used for topical anaesthesia of the airway. Lidocaine 2–4% applied to mucous membranes produces superficial anaesthesia in about one minute.^12^,^20^ The peak effect occurs within two to five minutes and the duration of action is 30–45min.^21^ The maximum safe dosage has generally been considered to be 3–4 mg. kg^−1^, although some recommend up to 6 mg./kg.^20^,^22^–^25^

**Advantages**: 1. ready availability; 2. relatively low CNS and cardiac toxicity; 3. quick onset; 4. clinically useful duration of action (30–60 mins for topical application and 1 to 2 hours after infiltration).

### Disadvantages:

1. Toxic plasma levels (>5 mcg. ml^−1^) can be reached when moderate amounts of high concentration solutions are used;
2. Caution must be used in patients with hepatic dysfunction.

Topical anaesthesia

**1. Topicalization** is the simplest method for anaesthetizing the airway. Topicalization of the airway is the spreading of local anaesthetic over a region of mucosa to achieve local uptake and neural blockade of that region

**Fig 4:**
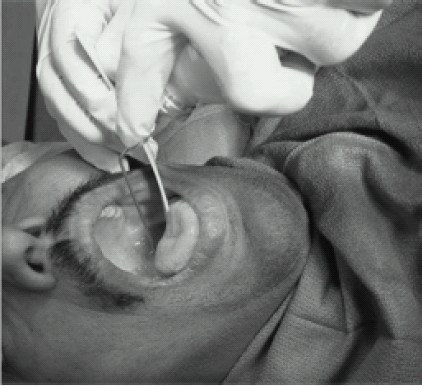
Local anaesthetic can be sprayed directly onto the Desired mucosa.

**2. Local anaesthetic can be sprayed**^12^ directly onto the desired mucosa.(Fig 4) (Pressurized solution of benzocaine, tetracaine, and butamben in a small canister, delivers a spray via a long spray nozzle pointed in the desired direction. (being oily its absorbed easily). Alternatively, a 10-mL syringe can be filled with lidocaine 2–4% and sprayed via a small-bore single or multiperforated catheter or the working channel of the fiberoptic bronchoscope.)

**Fig 5:**
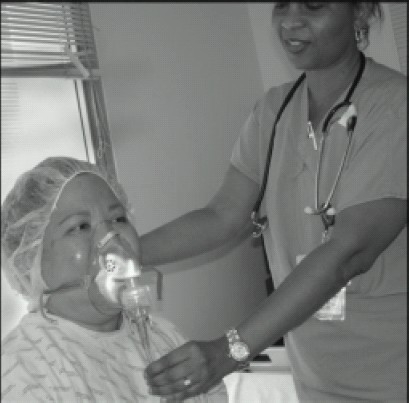
Nebulization of lidocaine 2–4% via face mask

**3. Nebulization** of lidocaine 2–4% via face mask or oral nebulizer for 15–30 minutes can achieve highly effective anaesthesia of the oral cavity and trachea for intubation.(Fig 5). The major advantage of this technique lies in its simplicity and lack of discomfort. In addition, very little working knowledge of the anatomy of the region is required for its successful implementation

**4. Atomization**^12^,^18^ is ideal for airway topicalization during nasotracheal intubations.

5. Density of anaesthesia is variable and often requires supplementation to facilitate intubation. Some patients may have intact cough reflex making intubatiuon difficult. Rate of onset is highly dependant on patient compliance. deep breaths may also lead to CNS depression in previously diseased patients.

6. Anaesthetic-soaked cotton pledgets^12^,^18^,^24^,^26^,^27^ or swabs can be applied to targeted mucosal surfaces for 5–15minutes to effect selective blockade of underlying nerves. These are soaked in either viscous or aqueous solutions of local anesthetic The cotton acts as a reservoir for the anesthetic agent, producing a dense block. This technique is especially effective in the nasal passages. Cotton-tipped swabs soaked in either lidocaine or cocaine and placed superiorly and posteriorly in the nasopharynx to block the branches of the ethmoidal and trigeminal nerves. Bilateral nasal administration of anaesthetic provides partial posterior pharyngeal anaesthesia by affecting the Sphenopalatine nerve fibers, thus diminishing the gag reflex.

7. Vasoconstrictors such as epinephrine (1:200, 000) or phenylephrine (0. 05%) can be added to the solution to

1. To prolong the duration of nerve blocks
2. Causes mucosal vasoconstriction, which improves visualization during the procedure and helps limit bleeding.

Topical nasal vasoconstrictors^28^–^30^

1. Neosynephrine

- Adults (~_12 yr): 0.25–0. 5%, 2–3 sprays in each nostril

- Children (6–12 yr): 0.25%, 2–3 sprays in each nostril

- Infants (<6 too): 0.16%, i-2 drops in each nostril

2 Xylometazoline

- Adults: 0.1%, 1–2 sprays or I metered dose spray or 2–3 drops in each nostril

- Children (>6 yr): 0.05%, 1–2 sprays or 2–3 drops in each nostril

- Children (>6 yr): 0.05%, 1 spray or I drop in each nostril

3. Oxymetazoline

- Adults and children >6 yr: 0.05%, 2–3 sprays or drops in each nostril

-Children (2–5 yr): 0.025%, 2–3 drops in each nostril

8. Gargling often does not cover the larynx or trachea adequately.

### Techniques for Block Individual Nerves of the Airway

Often more difficult to perform, and carry a higher risk of complications than the above mentioned methods. The common complications of nerve blocks are: bleeding, nerve damage, and intra-vascular injection.

### Nerve blocks

There are 3 blocks used for upper airway anesthesia:

1. Glossopharyngeal block – for oropharnyx.
2. Superior laryngeal block – larynx above the cords.
3. Translaryngeal block – larynx and trachea below the cords.

### Blockade of the Glossopharyngeal Nerve

It provides sensory innervation to the posterior third of the tongue, the vallecula, the anterior surface of the epiglottis (lingual branch), the walls of the pharynx (pharyngeal branch), and the tonsils (tonsillar branch)^31^

Facilitates endotracheal intubation by blocking the gag reflex associated with direct laryngoscopy as well as facilitating passage of a nasotracheal tube through the posterior pharynx. Glossopharyngeal nerve block is not adequate as a solo technique to facilitate intubation, but in combination with other techniques it is highly effective.

Blockade of the Glossopharyngeal Nerve

It can be blocked using one of three methods:

1. Topical spray application,
2. Direct mucosal contact of soaked pledgets, or
3. Direct infiltration by injection.

## Glossopharyngeal block

**Fig 6:**
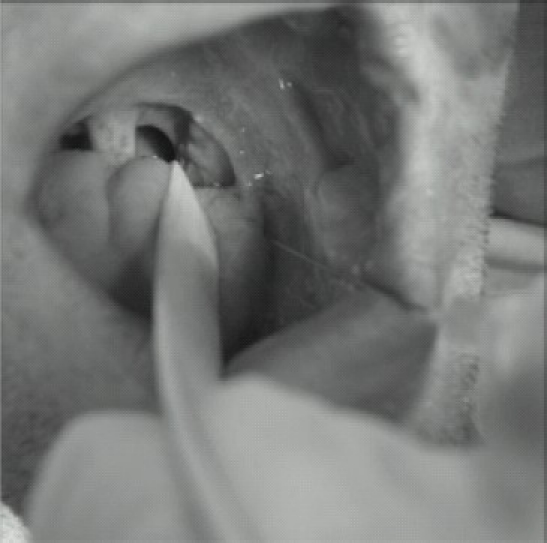
Glossopharyngeal block (Intraoral approach).

There are two way to approach:

1. Intra-oral – need enough mouth opening(Fig 6)

It is most easily blocked Intraorally by instilling 5 mL LA submucosally (by a 22-gaugue needle) at the caudal aspect of the posterior tonsillar pillar (where it crosses the palatoglossal arch).

**Fig 7:**
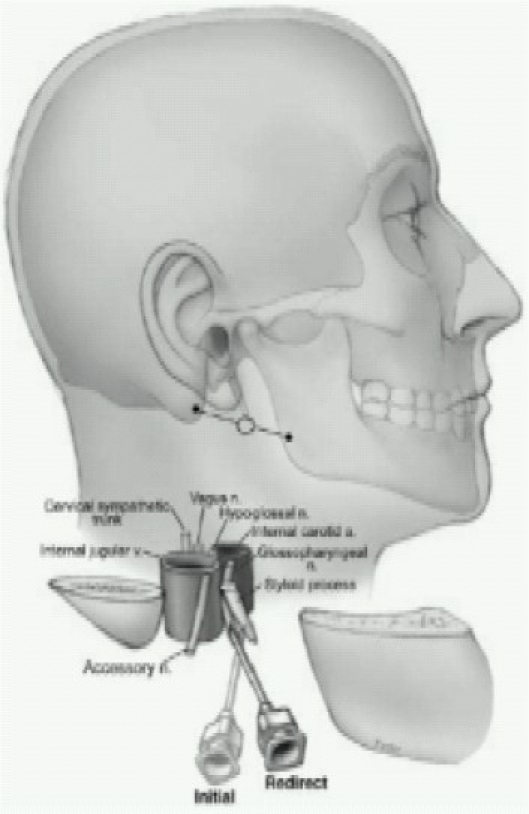
Glossopharyngeal block (Peristyloid approach)

2. Peristyloid – require the ability to distinguish the bony landmarks (Fig 7)

Patient is placed supine and a line is drawn between the angle of the mandible and the mastoid process. Using deep pressure, the styloid process is palpated just posterior to the angle of the jaw along this line, and a short, small-gauge needle is seated against the styloid process. The needle is then withdrawn slightly and directed posteriorly off the styloid process. As soon as bony contact is lost, 5–7 mL of local anesthetic solution are injected after careful aspiration for blood.

For both approaches, careful aspiration for blood must be carried out prior to injection (to prevent inadvertent intravascular injection) because the glossopharyngeal nerve is closely associated with the internal carotida. & palatoglossal arch is highly vascular and even a very small amount of local anesthetic can cause seizures.

Contraindicated in patients with coagulopathies or anticoagulation.

### Superior laryngeal block (Fig 8)

Internal branch^12^,^32^ (originates from the superior laryngeal nerve lateral to the greater cornu of the hyoid bone. The nerve should pass approximately 2–4 mm inferior to the greater cornu of the hyoid bone. where it pierces the thyrohyoid membrane and travels under the mucosa in the pyriform recess.) innervates the base of the tongue, posterior surface of the epiglottis, aryepiglottic fold, and the arytenoids^33^

**Fig 8:**
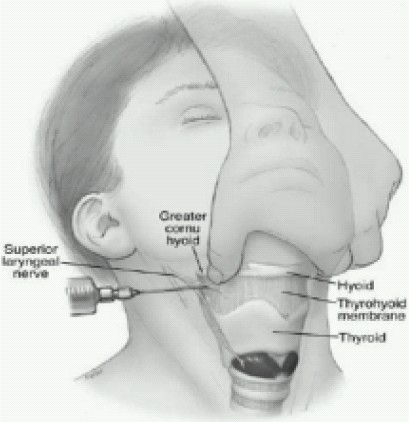
Superior laryngeal block

Blockade is usually inadequate as a solo technique for intubation. The superior laryngeal nerve can be blocked in the piriform fossa where it runs just deep to the mucosa by using Kraus or Jackson forceps to hold a cotton pledget soaked in lidocaine 2–4% against the mucosa for about 60 see^12^,^24^,^26^,^27^. Alternatively, this block can be performed using an external approach to the nerve as it penetrates the thyrohyoid membrane near the greater cornu of the hyoid^26^,^34^ Direct infiltration is accomplished by a 25G needle at the level of the thyrohyoid membrane inferior to the cornu of the hyoid bone^33^,^35^. A reliable block with a definite endpoint is effected by retracting the needle marginally after contacting the greater cornu and injecting2mLofLA (2%lidocaine) after negative aspiration in extended neck position.

The hyoid bone can be easily fractured if excess pressure is applied. 2 ml of LA should reliably bathe the internal branch of the superior laryngeal nerve, given at its proximity to the hyoid bone. If this volume is injected outside the thyrohyoid membrane, it is likely to block the external branch of the superior laryngeal nerve as well. Isolated external branch blockade may result in cricothyroid muscle weakness, which eliminates its function as an airway dilator. The motor input of the recurrent laryngeal nerve is spared, however, and therefore does not result in clinically significant change in laryngeal inlet diameters.

In case of contraindications, unwillingness or distorted anatomy noninvasive blockade can be accomplished by placing anesthetic-soaked cotton pledgets into the pyriform fossae bilaterally(for 10–15 min).

### Recurrent Laryngeal Nerve Block (Fig 10)

Recurrent laryngeal nerve provides sensory innervation to the trachea and vocal folds. Blockade facilitates comfortable passing of the endotracheal tube into the trachea.

**Fig 9:**
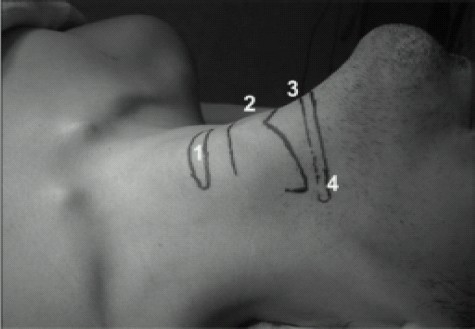
Normal surface anatomy of Larynx:1) cricoids cartilage 2) thyroid cartilage 3) hyoid bone 4) cornu of hyoid

**Fig 10:**
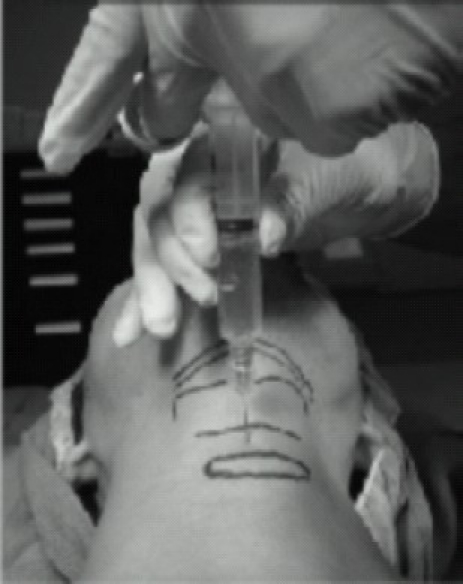
Recurrent Laryngeal Nerve Block Translaryngeal block of the recurrent laryngeal nerve at the level of the cricothyroidmembrane.

This nerve can be blocked by using topicalization techniques described previously.

Translaryngeal block of the recurrent laryngeal nerve is easily accomplished at the level of the cricothyroidmembrane. A 10-mL syringe with a 22- or 20-gauge needle is advanced until air is aspirated into the syringe. 4ml of LA (4%lidocaine) are then injected, inducing coughing that disperses the local anesthetic.

The recurrent laryngeal nerve can also be blocked by spraying local anesthetic via the injection port of the fiberoptic bronchoscope. Motor function remains completely unaffected. Direct infiltration of recurrent laryngeal nerve is contraindicated as it may cause upper airway obstruction since all motor supply to larynx except cricothyroid is by recurrent laryngeal nerve.

### Blockade of the Palatine Nerves

Nasal intubation requires blockade of the nasal passages.

Blockade of the greater and lesser palatine nerves blocks sensation to the nasal turbinates and posterior two thirds of the nasal septum. Topicalization of these structures is typically effective for intubation. Alternatively, the pterygopalatine ganglion can be blocked by passing a local anaesthetic-soaked cotton applicator along the upper border of the middle turbinate to the posterior wall of the nasopharynx, where it is left for 5–10 minutes. Transoral and percutaneous approaches to the pterygopalatine ganglion can be accomplished, but technical difficulty and an increased potential for complications preclude their routine use.^36^

### Blockade of the Anterior Ethmoid Nerve

The anterior ethmoid nerve innervates the remainder of the nasal passage. Anesthetic soaked cotton applicator is passed along the dorsal surface of the nose until the anterior cribiform plate is reached to achieve selective blockade after 5–10 minutes.

Theoretically, in high-risk situations, administration of local anaesthesia to the larynx and trachea may predispose to aspiration by obtunding protective air- way reflexes.^27^,^34^ However, these techniques have been used in these circumstances without associated aspiration,^30^,^37^,^38^ and the relative risk must be weighed against those associated with other airway management modalities. A common sense approach using good clinical judgement is mandatory

A combination of techniques is required to adequately anaesthetise upper airway structures for awake intubation. The widest coverage is provided by the inhalational technique. This technique, however, does not always provide a dense enough level of anaesthesia for all patients. Supplementation of this technique with any of the specific nerve blocks is an excellent way to accomplish efficacious anaesthesia for awake inubation.^39^ by laryngoscopic or fiberoptic method^40^.
